# A comparison of four different approaches to measuring health utility in depressed patients

**DOI:** 10.1186/1745-6215-14-S1-O26

**Published:** 2013-11-29

**Authors:** Nicholas Turner, John Campbell, Tim Peters, Nicola Wiles, Sandra Hollinghurst

**Affiliations:** 1 University of Bristol, Bristol, UK; 2 Peninsula Medical School, Exeter, UK

## Background

A variety of instruments are used to measure health related quality of life. Few data exist on performance and agreement of different instruments in a depressed population. The aim of this study was to investigate agreement between, and suitability of, the EQ-5D-3L, EQ-5D Visual Analogue Scale (EQ-5D VAS), SF-6D and SF-12 new algorithm for measuring health utility in depressed patients.

## Methods

The intraclass correlation coefficient (ICC) and Bland and Altman approaches were used to assess agreement. Instrument sensitivity was analysed by: plotting utility scores against one another; correlating utility scores and depressive symptoms (Beck Depression Inventory (BDI)); and using Tukey’s procedure. Receiver Operating Characteristics assessed instrument responsiveness to change. Acceptability was assessed by comparing completion rates.

## Results

The overall ICC was 0.57. Bland and Altman plots showed wide limits of agreement (except between SF-6D and SF-12 new algorithm). Utility score plots displayed ’ceiling-effects’ in the EQ-5D-3L and ’floor-effects’ in the SF-based instruments. All instruments showed a negative monotonic relationship with BDI, but the EQ-5D-3L and EQ-5D VAS could not differentiate between depression severity sub-groups. The SF-based instruments were better able to detect changes in health state over time. There was no difference in completion rates.

## Conclusions

There was a lack of agreement between utility scores generated by the different instruments. According to the criteria of sensitivity, responsiveness and acceptability, the SF-6D and SF-12 may be more suitable for the measurement of health related utility in a depressed population than the EQ-5D-3L, which is the instrument currently recommended by NICE.

